# DNA Sensors with Diamond as a Promising Alternative Transducer Material

**DOI:** 10.3390/s90705600

**Published:** 2009-07-14

**Authors:** Veronique Vermeeren, Sylvia Wenmackers, Patrick Wagner, Luc Michiels

**Affiliations:** 1 Biomedical Research Institute, School for Life Sciences, Hasselt University and Transnationale Universiteit Limburg, Agoralaan, Bldg. C, B-3590 Diepenbeek, Belgium; E-Mail: veronique.vermeeren@uhasselt.be; 2 Institute for Materials Research, School for Life Sciences, Hasselt University and Transnationale Universiteit Limburg, Wetenschapspark 1, B-3590 Diepenbeek, Belgium; E-Mail: patrick.wagner@uhasselt.be (P.W.)

**Keywords:** DNA, single nucleotide mismatch, aptamers, bioconjugates, biosensors, impedance sensors, SPR, fluorescence sensors

## Abstract

Bio-electronics is a scientific field coupling the achievements in biology with electronics to obtain higher sensitivity, specificity and speed. Biosensors have played a pivotal role, and many have become established in the clinical and scientific world. They need to be sensitive, specific, fast and cheap. Electrochemical biosensors are most frequently cited in literature, often in the context of DNA sensing and mutation analysis. However, many popular electrochemical transduction materials, such as silicon, are susceptible to hydrolysis, leading to loss of bioreceptor molecules from the surface. Hence, increased attention has been shifted towards diamond, which surpasses silicon on many levels.

## 1. Introduction

The transition from ‘macro’ to ‘micro’ and recently to ‘nano’ has been noticeable in many scientific fields. In biology, the evolution towards research into the micro- and nanobiotic world has led to the identification of many disease-related pathogens. In the technological field, the progression from electronics into micro- and nano-electronics has enabled the miniaturisation of computers. Combining the enormous advances in biology with those attained in micro- and nano-electronics has laid the groundwork to study biology on a single molecule level and to embark into the ‘nano’-world. In this context, nanometer-scale biomolecules have become an integrated component of electronic devices designed for early, reliable and affordable detection of target molecules. Hence, the development of these biosensors has been a pivotal point in the evolvement of this new ‘bio-electronics’ field. In the next paragraph, we will describe the different parts in biosensor construction. For more detail, the reader is referred to the work in [1].

### 1.1. Biological Receptor Molecules

The type of biological receptor molecule determines the bio-selectivity, and hence the type of biosensor. Enzymes recognise their substrates and will convert it to a reaction product. The associated biosensors are termed ‘catalytic or enzyme biosensors’. Other molecules identify their targets by forming an affinity-complex governed by hydrogen (H) bonds, hydrophobic interactions, electrostatic forces, and ‘van der Waals’ forces. Seperately, these non-covalent bonds are very weak. However, the presence of many of these bonds in one affinity interaction can cause a very strong association between target and receptor. Biosensors with a selectivity based on the formation of an affinity-complex are called ‘affinity-based biosensors’. One of the most common biomolecules that recognises its target through such an affinity-complex, is the antibody. The affinity-based biosensor subgroup containing immobilised antibodies is termed ‘immunosensor’. The receptor can also be a single-stranded DNA (ssDNA) molecule, yielding a ‘DNA-based biosensor’. Furthermore, membrane receptors and even whole cells can serve as the biological part of a biosensor.

### 1.2. Attachment of Biological Receptor Molecules

Detection of target molecules and signal transduction usually must occur at a liquid-solid interface. For this reason, the efficient immobilisation of the biological receptor molecule onto the transducer is a crucial point for the performance of a biosensor. The benefit of the immobilisation technique must be two-fold: it should result in a stable layer of biomolecules and these biomolecules should retain their biological activity. The available immobilisation techniques can be grouped into two categories: non-covalent binding, by physical adsorption, and covalent binding.

#### 1.2.1. Non-Covalent, Physical Adsorption

Generally, physical adsorption results in significant losses of biomolecules from the surface because of the rather weak bonds involved to immobilise them. Moreover, physical adsorption leads to random orientations of the molecules, more often than not rendering the part that engages in target recognition inaccessible, thereby lowering device sensitivity. However, some non-covalent binding approaches do yield a firmly immobilised and well-oriented biomolecule layer, such as streptavidin-biotin interactions. Streptavidin-modified surfaces bound with biotinylated biomolecules result in the strongest non-covalent bond known. The streptavidin-biotin complexes are also extremely stable over a wide range of temperatures and pH values [2].

#### 1.2.2. Covalent Binding

Covalent attachment of biomolecules to solid surfaces is the immobilisation technique of choice for biosensor fabrication. It results in a stable and long-term modification of the substrate with oriented biomolecules. The surface of the transducer can be modified to present desired functionalities (-NH_2_, -COOH, -SH, …). Concerning the fabrication of DNA-based biosensors, ssDNA can subsequently be coupled to these functional groups through its own range of possible end modifications, ensuring a covalently bound DNA molecule that is available for hybridisation. DNA molecules containing carboxylic acid (COOH)-functionalities can be bound with amino (NH_2_)-groups and form peptide bonds (R-CO-NH-R). When they are modified with a thiol (SH)-group, linking with a COOH-functionality will yield a thiol ester (R-CO-S-R-), while binding with another SH-group will result in a disulfide bridge (C-S-S-C). SH-modified DNA can also react with the double bond of a maleimide compound [(CH)_2_-(CO)_2_-NH)] to form a stable carbon-sulfur bond (C-S).

Antibodies and enzymes, being proteins, contain NH_2_-groups, COOH-groups and SH-groups. Thus, they can also easily be covalently bound to NH_2_-, COOH- and SH-modified surfaces. Proteins are also often coupled to surfaces using the maleimide chemistry described above. Moreover, a widely used approach for efficient and well-oriented antibody attachment makes use of protein A or G. The latter proteins are immobilised onto the solid substrate, and form a linker layer for the covalent attachment of antibodies with their antigen-recognising Fab fragments protruding outwards.

Also, the capability of many organic molecules, such as alkylsilanes, to spontaneously self-assemble into monolayers on several solid substrates provides a very useful tool to engineer a desired environment on the transducer surface. These self-assembled monolayers (SAMs) have a well-defined and ordered molecular architecture. The alkoxysilanes are widely used for this purpose. In alkoxysilanes, two classes of moieties are attached to the silicon (Si) atom. There is an organic moiety that can be a carbon chain (alkyl), an aromatic group (aryl), an organofunctional group (-NH_2_, -COOH, -SH, …) or a combination of these. Alkyl and aryl silanes can form hydrophobic coatings and act as water repellants, while organofunctional silanes can react with other molecules to be attached in the ways described above. The alkoxy moiety usually consists of three methoxy or ethoxy groups. First, the two lateral alkoxy groups hydrolyse and cause condensation with other alkoxysilanes in a lateral assembly. The third alkoxy group first forms hydrogen bonds with and then covalently binds to sites on the surface while liberating water [1].

### 1.3. Types of Transducers

Many kinds of substrate materials can be used for the immobilisation of biomolecules in biosensor construction. Common ones are polystyrene [3], gold (Au) [4], silicon (Si) [5] and silicon oxide, beads [6], and recently, diamond. When devising a biosensor, care must be taken in choosing the substrate material. This decision depends strongly on the signal generating mechanism one has in mind. Not all substrate materials are suitable for every type of transduction. So, next to the type of biological receptor molecule, the type of transducer material is also determined by the type of biosensor. When envisaging an optical target detection, such as fluorescence or chemiluminescence, it is beneficial to use substrates with a low background luminescence. The conductivity of the substrate is of negligible importance so glass, plastics or Si are often used.

For electrochemical biosensors, however, the importance of the electrical properties of the transducer rises drastically. Materials such as Au, Si, germanium (Ge), Pt, etc. are optional. However, the bonds between metal electrodes such as Au and Pt and biomolecules are sensitive to oxidation, causing substantial loss of target receptors from the surface. Moreover, their metallic conductivity makes them perpetually conducting, hampering and even inhibiting the control of their electronic properties. For this reason, increased attention is being addressed towards semiconductor materials, such as Si and Ge. Nevertheless, biofunctionalised Si surfaces are very susceptible to hydrolysis, also leading to significant loss of bound biomolecules, decreasing their sensitivity over time [7,8]. Recently, diamond has attracted much attention as a possible alternative semiconductor material. It surpasses the more generally used semiconductor electrode materials such as Si and Ge at many levels. An overview of the physical properties of diamond in comparison to Si and Ge is given in Table 1 [9,10].

The large bandgap of diamond will ensure that electrons will not easily enter the conduction band, compromising its intrinsic insulating properties. At the same time, UV-radiation, with an energy ranging from a few to about 100 eV, can make valence electrons jump this gap, allowing applications as UV-photosensors. This bandgap feature also explains the high resistivity and high breakdown voltage of diamond. Its high thermal conductivity, combined with its low thermal expansion, makes diamond an ideal material to be used as a heat sink in high-powered devices without the risk of being thermally deformed. Also, diamond can function as a dielectric in capacitors. Its low dielectric constant allows diamond to withstand high electric fields, reflected in its high breakdown voltage. The next section elaborates on these appealing properties and the applications of diamond in the bio-electronics field.

## 2. The Role of Diamond in Biosensors

The advances in biosensor development ultimately depend on the perpetual search for optimal transducer materials, allowing rapid, sensitive and selective biological signal detection and translation. Candidate materials must possess a number of important characteristics for them to be considered as transducers. First of all, they need to be able to undergo biofunctionalisation. This factor has been investigated and achieved for several substrates, such as latex beads [6], polystyrene [3], carbon electrodes [11], Au [4] and glass, as previously mentioned.

Secondly, the substrates need to be reasonably flat and homogeneous, and the resulting bio-interfaces should be able to be manufactured with a high reproducibility. These requirements eliminate the possible use of beads, polystyrene and glass.

Finally, the bio-interfaces will be integrated into micro-electronics, requiring the materials to be compatible with micro-electronic processes, as is the case with carbon electrodes, Au and Si. Unfortunately, these are not chemically stable and the bio-interfaces degrade upon contact with aqueous electrolytes [8]. Diamond has become an attractive alternative candidate for its use as a transducer material in bio-electronics. It is the only material that is compatible with processes applied in micro-electronics that does not show any degradation in electrolytes, even at fairly high potentials. The following sections will elaborate more on these appealing features as well as on the classification of diamond into different subtypes, not all of them being appropriate for biosensor development.

### 2.1. Classification of Diamond

#### 2.1.1. Natural Diamond

**Figure 1.**
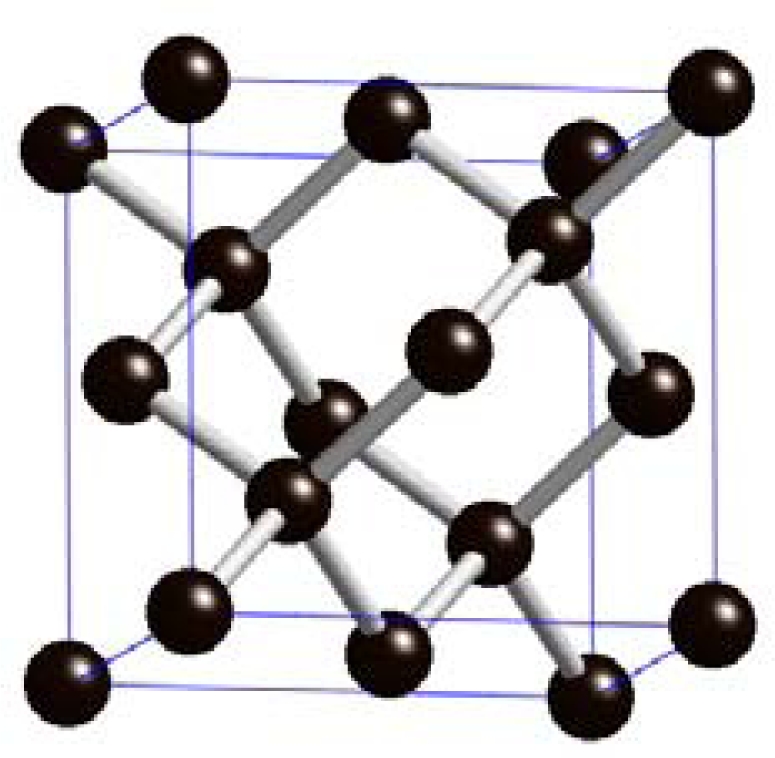
Schematic diagram of the lattice structure of diamond, showing the tetrahedral orientation of each C atom.

In diamond, each carbon (C) atom forms four single sigma (σ) bonds, each consisting of a sp^3^-hybridised orbital, with four neighbouring C atoms in a tetrahedral-structure. Each bond is at an angle of 109°28′ to the adjacent bond. Each tetrahedron is associated with four other tetrahedrons, forming a very stable and tightly bound covalent lattice or crystal structure. This explains the extreme hardness of diamond, one of its unique properties that will be described further in Section 2.2 [12]. The diamond lattice is shown in Figure 1.

The classification system of natural diamond is based on the presence of nitrogen (N) impurities in the lattice structure, which can easily be assessed using Infrared (IR) absorption spectroscopy. The presence of these N impurities disturbs the perfect crystal structure of diamond. This causes certain vibrational modes, such as one-phonon processes, to occur upon illumination with IR light that usually do not take place because of the lattice symmetry. About 78% of natural diamonds contain enough N to be detectable by IR absorption spectroscopy [13,14].

##### Type I diamonds

Type I diamonds contain a lot of N, roughly >10^17^–10^18^ atoms·cm^−3^. They are further divided into subdivisions Ia and Ib [13,14].

Type Ia is the most common type of natural diamond. 78% of all natural diamond is classified as type Ia. They usually contain clusters of N. Because these diamonds absorb blue light, they can have a pale yellow or brown colour.

If the N atoms are atomically dispersed throughout the carbon lattice, then the diamond is said to be a type Ib diamond. These diamonds absorb green light as well as blue light, and have a darker colour than type Ia diamonds. Depending on the precise concentration and spread of the N atoms, these diamonds can appear deep yellow, orange, brown or green. The donor energy level of the substitutional N is deep in the bandgap of diamond, making it unsuitable for electronic applications. Less then 0.1% of natural diamonds belong to type Ib.

##### Type II diamonds

Type II diamond contains only small amounts of N, roughly <10^17^–10^18^ atoms·cm^−3^. Hence, they do not show any N-related IR absorption features. They are also further divided into subdivisions IIa and IIb [13,14].

Type IIa diamonds are considered the purest of all natural diamonds. They do not contain any impurities at all, hence they are optically the most transparent. ∼22% of natural diamonds belong to type IIa, and are very much appreciated as a gemstone.

Type IIb diamonds contain no N, but they do contain boron (B). For this reason, they are naturally occurring p-type semiconductors (see Section 2.2.1), containing a maximum B concentration of 10^17^ atoms·cm^−3^. They absorb red, orange and yellow light, and therefore usually appear to be blue. They are also rare, making up 0.1% of all natural diamonds.

#### 2.1.2. Synthetic Diamond

Since the useful mechanical, electronical and chemical properties of diamond, which will be discussed in Section 2.2, became known, numerous attempts were made to synthesise diamonds. It was hoped this would alleviate the high prices, scarcity and the inability to tailor the naturally occurring diamonds, limiting their use for technological applications. Moreover, the impurities that were present in 78% of all natural diamonds influenced and deteriorated the diamond properties, restricting the use of natural diamonds in technology. In the course of history, two main procedures have been developed to synthesise diamonds.

High Pressure–High Temperature (HPHT) is a technique that mimics the natural process that produces diamond from graphite. The technique has been perfected since 1955, providing a reliable way to produce synthetic diamonds [15]. However, since the procedure requires a metal solvent, traces in the diamond end product are not rare, leading to application-limiting defects in the diamonds produced.

A more user-friendly and flexible method, allowing operation under lower pressure and temperature regimes, was obtained with the development of Chemical Vapour Deposition (CVD). Activation of a C-containing precursor gas mixture, most often consisting of methane (CH_4_) and hydrogen gas (H_2_), leads to the deposition of diamond onto a substrate [16]. With this technique, it became possible to deposit thin films of diamond on a variety of substrates within reasonable time scales [17].

Two basic forms of diamond can be synthesised, depending on certain diamond growth parameters: single-crystalline diamond (SCD) and polycrystalline diamond (PCD). The latter can be further subdivided according to the size of the crystals it is composed of: microcrystalline diamond (MCD), nanocrystalline diamond (NCD) and ultrananocrystalline diamond (UNCD).

##### Single-crystalline diamond (SCD)

**Figure 2.**
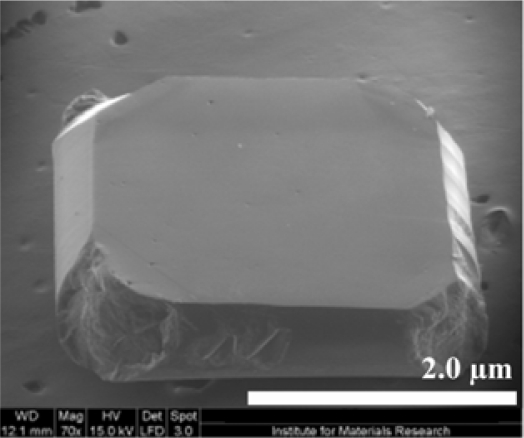
SEM image of a 600 μm thick freestanding homo-epitaxial SCD film grown at a 10% CH_4_/H_2_ ratio removed from the substrate by laser cutting *(IMEC, Belgium, Wide Band Gap Materials)*.

SCD is grown using HPHT. Thin slices of natural or previously grown SCD are used as substrates or seed material for the graphite or feed material, making this a rather expensive procedure. Because there will be no lattice mismatch between the SCD seed material and the newly deposited diamond, a SCD layer will be formed. The diamond is said to have been grown homo-epitaxially. SCD growth has been optimised over the years to yield atomically smooth and defect-free diamond films [18]. A Scanning Electron Microscope (SEM) image showing a SCD film is presented in Figure 2.

##### Polycrystalline diamond (PCD)

###### • Microcrystalline diamond (MCD)

MCD is synthesised by CVD on substrates with a lattice mismatch using and is therefore said to be grown hetero-epitaxially. The substrate is pre-treated by scratching the surface with diamond powder to roughen the surface and act as seeds, or nucleation sites, for diamond growth [16]. The growth starts simultaneously at every nucleation site, forming columnar structures, called grains, that increase in diameter as growth proceeds. These grains eventually coalesce, leading to a surface consisting of μm-range grains delineated by their grain boundaries. These are rich in graphitic sp^2^-inclusions and defects.

**Figure 3.**
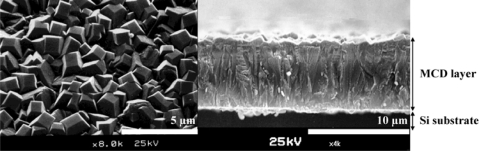
SEM images of a MCD film on Si. In a topographical view, the μm-sized grains on the surface are clearly visible (left panel). The cross-section shows the columnar growth nature of these grains (right panel) *(University of Bristol, UK, CVD Diamond Film Group)*.

Depending on certain growth parameters, the orientation of the facets of the grains with respect to the surface can be controlled. MCD shows significant roughness, in the range of 1 to several tens of μm. For this reason, MCD is usually polished to obtain a smooth surface. However, this causes a thin and damaged surface, deteriorating its potential use in electronic applications [8]. Figure 3 shows a SEM image of a MCD film.

###### • Nanocrystalline diamond (NCD)

**Figure 4.**
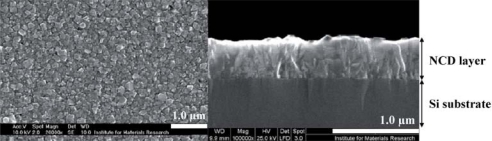
SEM images of a NCD film on Si. In a topographical view, the nm-sized grains on the surface are clearly visible (left panel). The cross-section shows the thinner diamond layer on top of the Si (right panel) *(Institute for Materials Research, Belgium).*

NCD is grown identically to MCD, yet the density of nucleation sites is made higher by increasing the concentration of the diamond powder during substrate pre-treatment. The result is a diamond film consisting of smaller (in the nm-range), and hence more, grains. This increases the amount of amorphous C and defects present in the grain boundaries. The grains facets are randomly oriented, resulting in a ‘cauliflower’-like surface structure. The maximum attainable NCD film thickness is limited, since longer growth times cause the columnar crystals to become ever larger in diameter, eventually reaching MCD status. NCD and MCD are transparent and can be made semiconducting [19]. A SEM image is displayed in Figure 4.

###### • Ultrananocrystalline diamond (UNCD)

While the other PCD films are grown in a H-rich environment, UNCD is synthesised in an Argon-atmosphere. Instead of substrate nucleation before diamond growth, there is continuous re-nucleation during synthesis.

**Figure 5.**
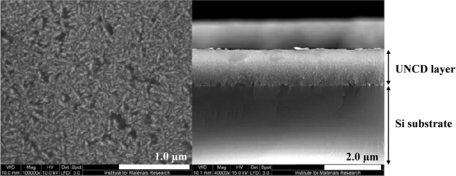
SEM images of a UNCD film on Si. In a topographical view, the even smaller grains on the surface are clearly visible (left panel). The cross-section is shown in the right panel *(Institute for Materials Research, Belgium).*

This leads to even smaller grain sizes and to an even higher amount of non-diamond C phases in UNCD than in NCD, decreasing the optical transparency of UNCD films with respect to NCD. Also, UNCD is not semiconducting, but shows metallic conductivity governed by the grain boundaries at the surface [19]. This could create some difficulties in biosensor implementation. A UNCD SEM image is presented in Figure 5.

### 2.2. Properties of Diamond

#### 2.2.1. Electronic Properties

##### Large electrochemical potential window

**Figure 6.**
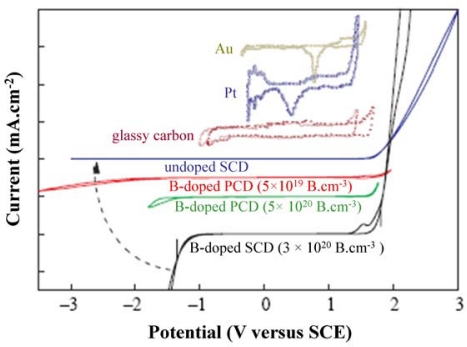
Cyclic voltammograms comparing various electrode materials. The graphs are shifted vertically for comparison. Two B-doped PCD films, B:PCD(NRL) containing 5 × 10^19^ B·cm^−3^ and B:PCD(USU) containing 5 × 10^20^ B·cm^−3^ [20], are compared with a B-doped SCD film, B:(H)SCD, containing 3 × 10^20^ B·cm^−3^, and with an undoped SCD film, (H)SCD. Voltammograms of alternative electrodes such as Au, Pt and glassy carbon are also added for comparison [8].

Compared with other commonly used electrode materials, such as Pt, Au and glassy carbon, diamond has a significantly larger electrochemical potential window. As determined by cyclic voltammetry (CV, see Section 3.1.1), the voltages at which oxidation (O_2_ evolution) and reduction (H_2_ evolution) occur are well separated by several volts. Oxidation reactions, occurring at a positive potential of ∼1.8 V, produce positive currents. Reduction reactions produce negative currents and occur at different negative potentials for different kinds of diamond. For instance, by increasing the B-doping level from intrinsic (undoped) diamond to metallic diamond (>10^20^ atoms·cm^−3^), the onset of H_2_ evolution can be switched on. Moreover, the background currents within this potential window are considerably smaller as compared to glassy carbon, Pt and Au. This is shown in Figure 6 [8].

##### Hydrogen (H)-induced surface conductivity

When H-terminated diamond is brought into contact with an electrolyte, a phenomenon called ‘transfer doping’ occurs. When the highest valence band energy level, or valence band maximum (VBM) of a material is higher than the chemical potential of the electrolyte (μ) in contact with that material, electrons can transfer from the valence band into the electrolyte, creating positively charged holes in the valence band. These holes in the valence band cause surface conductivity. However, for most semiconductors, such as Si and Ge, their VBM is lower than the chemical potential of the electrolyte, μ.

**Figure 7.**
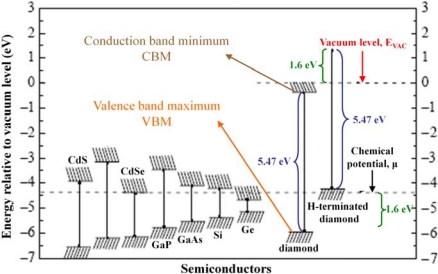
Energies of the valence and conduction band edges of several conventional semiconductors, including H-terminated and as-grown diamond relative to the vacuum level, E_VAC_. The lower dashed line represents the chemical potential, μ, for electrons in an acidic electrolyte under the conditions of a standard hydrogen electrode [21].

H-termination of diamond creates a dense surface dipole layer because of the polar covalent bonds between C and H. The energy of this dipole varies over 1.6 eV, shifting all energy levels in the valence and conduction band of diamond upwards with this value. Figure 7 shows an overview of the energies of valence band and conduction band edges of several semiconductors, among which as-grown and H-terminated diamond.

**Figure 8.**
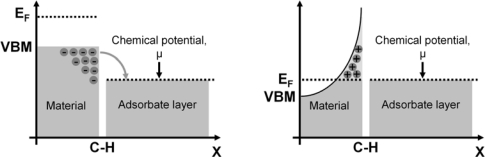
Schematic diagram of the diamond/electrolyte interface. Under non-equilibrated conditions (left panel), electrons from the valence band of the diamond tunnel into empty electronic states of the electrolyte. This continues until the E_F_ in the diamond and μ of the electrolyte align and reach thermodynamic equilibrium (right panel) [8].

Through the H-termination, the valence band energy level now surpasses the chemical potential of electrolytes, μ, allowing electrons to transcend into the electrolyte until the Fermi level (E_F_) of the diamond and the chemical potential of the electrolyte, μ, align and reach thermodynamic equilibrium. This forms a hole accumulation layer in the diamond. This is illustrated in Figure 8.

**Figure 9.**
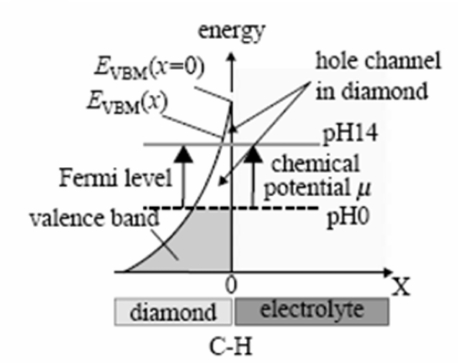
E_F_ and μ alignment at the diamond/electrolyte interface at pH 0 and pH 14 [8].

As the chemical potential of electrolytes, μ, is pH-dependent, the surface conductivity of H-terminated diamond also varies, as shown in Figure 9. It can be predicted by the Nernst equation, showing a pH-dependency of 55 mV/pH [21–23].

##### Diamond doping

Intrinsic or undoped diamond has a very wide bandgap of 5.47 eV at room temperature, giving it near insulator-like characteristics. Diamond can be doped, however, into a semiconductor by the introduction of impurity atoms into the carbon lattice. As already mentioned, the major interest in semiconductors lies in the ability to control current flow through them by exposure to an external energy source. Metals do not offer that option, since they almost always conduct electricity. Two types of diamond doping exist: p-type doping and n-type doping [8].

###### • p-type doping

Introduction into diamond of group III impurity atoms, for instance B atoms, results in p-type doping. In the diamond lattice structure, each C atom has four electrons in its outer, valence shell, that are shared with four other C atoms. The valence band, now containing eight electrons per C atom, is completely filled, forming a very stable crystal. B has only three electrons in its valence shell. When B is introduced into the lattice, an electron deficiency, or a positively charged hole, is created in the energy level directly above the valence band of diamond, called the acceptor level. This hole can be filled by the movement of an electron from the valence shell of a neighbouring C atom into the hole of the B atom. B is thus called an acceptor atom. By filling the electron vacancy, a new hole is now created in the valence shell of the C atom that donated the electron, which itself can be filled by another neighbouring electron. The result is a movement of positively charged holes in the valence band of diamond. These holes are thus called the majority charge carriers.

In p-type diamond, the acceptor level lays 3.60 eV above the VBM, thus being 1.87 eV below the conduction band minimum (CBM). This gap is too deep to be crossed at room temperature, a regime that has the most bio-electronic relevance. However, when the diamond is metallically doped with B, typically corresponding to 10^20^ atoms·cm^−3^, enough holes will exist in the valence shell of diamond to propagate a current in the valence band without the need for thermal activation.

###### • n-type doping

**Figure 10.**
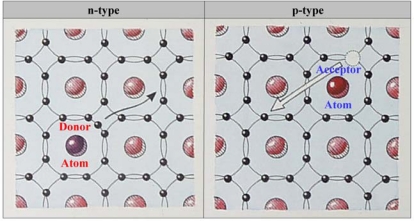
Schematic diagram of an n-type and p-type semiconductor material at the atomic level.

Introduction into diamond of group IV impurity atoms, such as phosphorous (P) atoms, results in n-type doping. P has five electrons in its valence shell. When P is incorporated into the diamond lattice, a situation is created where additional free electrons are supplied to the diamond lattice. Hence, P is called a donor atom. These electrons are very loosely bound in the diamond crystal, and occupy an energy level directly (0.6 eV) below the conduction band, termed the donor level. Hence, only a small amount of energy is needed to promote excitation into the conduction band. The result is a movement of negatively charged electrons in the conduction band of diamond. These electrons are the majority charge carriers. In 1997, Koizumi and coworkers were the first to succeed in producing n-type doped SCD using phosphine [24]. The difference between a p-type and an n-type semiconductor is graphically presented in Figure 10.

#### 2.2.2. Physical Properties

##### Hardness

Diamond is the hardest material found in nature. It scores a ‘10’ on the Mohs scale. The Mohs scale, developed in 1822 by the Austrian Friedreich Mohs, ranks ten minerals for their ability to scratch another mineral in the series. Another more quantitative scale, the Knoop scale, classifies materials according to the force needed to make indentations in them with a diamond. Again, diamond ranks the highest. Diamond’s hardness is not a constant quantity but varies even within a single diamond.

Because of the extreme hardness of diamond, tools can be coated with diamond for their use in the most important and demanding mechanical applications, such as sawing, drilling, cutting and polishing. Diamond coatings enhance their performance and prolong their lifetime. However, metals like manganese, iron, cobalt, nickel and Pt act as a solvent for carbon. For this reason, industrially important materials cannot be processed with diamond-coated tools [25,26].

##### Thermal conductivity

Thermal conductivity indicates the ability of a material to conduct heat. In metals, thermal conductivity parallels electrical conductivity, as electrons transfer not only current but also heat energy. In other non-metal materials, this relationship between electric current and heat transfer fades. Phonons, quantised vibrational modes in a crystal lattice, are the major carriers of heat in these substances. Diamond has a very high thermal conductivity of 9–23 W·cm^−1^·K^−1^, exceeding that of, for instance, Si (1.68 W·cm^−1^·K^−1^), copper (Cu, 4.01 W·cm^−1^·K^−1^), silver (Ag, 4.29 W·cm^−1^·K^−1^) and Au (3.18 W·cm^−1^·K^−1^). Because atomic vibrations are controlled by temperature, thermal conductivity is usually temperature-dependent. This makes diamond an important material to be used in heat management. It can be used as a heat sink in computers, lenses, laser diodes, high-power integrated circuits and laser windows (see Section 1.3, Table 1) [25].

##### Optics

Diamond is transparent over a large range of wavelengths [from the ultraviolet (UV) to the far-IR]. Moreover, its large bandgap (5.47 eV) prevents thermally generated conduction at elevated temperatures. Therefore diamond remains transparent even at very high temperatures and radiation intensities. Diamond is thus an ideal material for optical applications, and can be used as X-ray and UV-transparent windows, and X-ray dosimeters.

The sparkling of a diamond is caused by an optical phenomenon called Total Internal Reflection (TIR). The light undergoes many internal reflections inside the diamond before it exits. When light crosses a boundary from a medium with a higher to one with a lower refractive index, the light will be partially refracted at the boundary surface, and partially reflected. At a certain critical angle, light is refracted such that it travels along the boundary. When the incidence angle is greater than the critical angle, all of the light will be reflected. This is called TIR. Total Internal Reflection Fluorescence (TIRF) spectroscopy makes use of this principle to study events at or close to the interface of two different media, for instance the analysis of cells on a coverslip in biological applications. The higher the refractive index of the material at the side of the incoming light beam with respect to the refractive index of the material at the other side of the boundary, the lower the critical angle at which TIR is obtained, and facilitating TIRF applications. The refractive index of diamond is 2.4, leading to a critical angle of 24.6°. This is much lower than for glass, having a refractive index of 1.5, and hence a critical angle of 40.5° [25].

#### 2.2.3. Biochemical Properties

**Figure 11.**
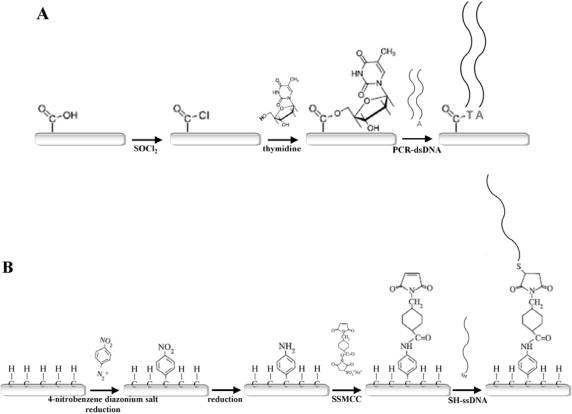

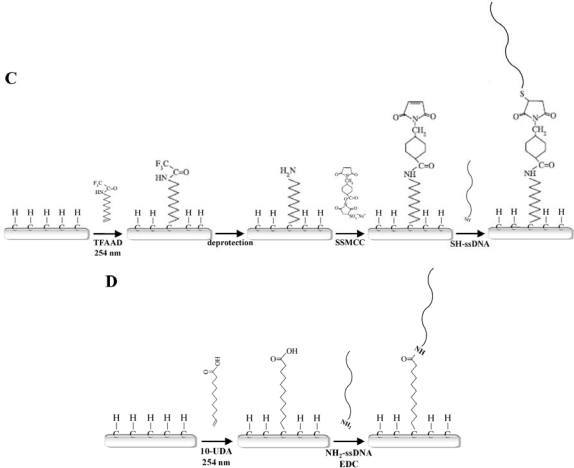
Different reaction mechanisms for the covalent attachment of DNA to diamond. A: Reaction process used by Ushizawa and coworkers for the covalent attachment of PCR-amplified dsDNA to thymidine-modified diamond powder [28]. The surface of the diamond powder was oxidised and the carboxyl groups were subsequently modified with thymidine. DNA molecules generated through PCR amplification could be covalently ligated to the thymidine-modified surface. B: Reaction process used by Wang and coworkers for the covalent attachment of thiolated ssDNA to aminophenyl-modified p-type SCD [29]. The SCD was treated with a diazonium salt, which was subsequently reduced to nitrophenyl and attached to the SCD. The nitrophenyl groups were reduced to aminophenyl groups, and the resulting NH_2_-modified SCD was modified with the cross-linker sulphosuccinimidyl-4-(N-maleimido-mehyl)cyclohexane-1-carboxylate (SSMCC). Thiol (SH)-modified ssDNA could then be linked to the COOH-moiety of SSMCC. C: Reaction process used by Yang and coworkers for the covalent attachment of thiolated ssDNA to photochemically activated NCD [7]. H-terminated NCD surfaces were covalently modified with trifluoro-acetamide acid (TFAAD) under UV-illumination. The top trifluoro-acetic acid groups at the NCD surface were removed, forming NH_2_-modified NCD surfaces, to which SSMCC was bound. SH-modified ssDNA could then be linked to the COOH-moiety of SSMCC. D: Reaction process used by our group for the covalent attachment of aminated ssDNA to photochemically activated NCD [30,31]. H-terminated NCD surfaces were covalently modified with 10-unedecenoic acid (10-UDA) under UV-illumination. NH_2_-modified DNA was then covalently bound to these COOH-groups through an amide bond via 1-ethyl-3-(3-dimethylaminopropyl)-carbodiimide (EDC).

Another appealing feature of diamond, in addition to the ones already described, is that the material is chemically inert. It does not degrade upon contact with aqueous electrolytes, as is the case with other well-known semiconductors used in the field of bio-electronics, such as Si. However, this inertness could be an obstacle to its use in biosensor-development, since biofunctionalisation cannot be achieved at an unreactive surface. Fortunately, in 2000, Takahashi and coworkers accomplished the activation of H-terminated diamond surfaces through a photochemical chlorination/amination/carboxylation [27], thereby. circumventing the barrier of diamond inertness, and paving the way towards the further modification of diamond with biomolecules, such as DNA and proteins (enzymes and antibodies). Together with the ability to be biofunctionalised, diamond is biocompatible, allowing for future *in vivo* electronic applications. In the next section, a summary of the most important biofunctionalisation routes are given which are also graphically depicted in Figure 11 [8].

##### Chemical biofunctionalisation

Ushizawa and coworkers reported the wet-chemical modification of diamond powder (1–2 μm) with thymidines [28]. First, the surface of the diamond powder was oxidised to its surface oxides [carboxylic acid (COOH), hydroxyl (OH), acid anhydride] by immersion into a heated mixture of sulphuric acid (H_2_SO_4_) and nitric acid (HNO_3_). Next, the carboxylated diamond was treated with thionyl chloride (SOCl_2_) and thymidine, resulting in a thymidine-modified diamond surface. DNA molecules generated through Polymerase Chain Reaction (PCR) amplification could be covalently attached to the thymidine-modified surface via a simple ligation reaction. PCR has the interesting characteristic of adding an adenine (A) base to the 3′ end of each amplified DNA molecule. These 3′A-overhangs were exploited in the ligation to the thymidine-modified substrate. Diffuse Reflectance Infrared Fourier-Transform spectroscopy (DRIFT) was used to verify the presence of DNA on the surface. A summary of their reaction process is given in Figure 11A. Though an attractive procedure, the modification process is tedious, labour-intensive, and multistep, and could not be reproduced on CVD diamond films.

##### Electrochemical biofunctionalisation

SCD of p-type nature has been covalently modified with DNA molecules through an electrochemical procedure by Wang and coworkers They used a three-electrode configuration with a SCD working electrode, a Pt counter electrode and a silver/silver chloride (Ag/AgCl) reference electrode. The p-type SCD working electrode was treated with the diazonium salt 4-nitrobenzene-diazonium tetrafluoroborate. This salt was reduced in acetonitrile to nitrophenyl using CV and attached to the SCD surface in a nitrogen gas (N_2_)-purged glove-box. The nitrophenyl groups were subsequently reduced to aminophenyl groups, resulting in a NH_2_-modified SCD surface. This NH_2_-modified SCD could then be modified downstream with the heterobifunctional cross-linker molecule sulphosuccinimidyl-4-(*N*-maleimidomethyl)cyclohexane-1-carboxylate (SSMCC). The *N*-hydroxy-succinimide (NHS)-ester group of SSMCC reacts with the NH_2_-groups on the NCD to form amide (NH) bonds. SH-modified ssDNA could then be linked to the COOH-moiety of SSMCC at room temperature, resulting in a covalent bond. DNA functionality was confirmed with fluorescent hybridisation detection [29]. However, this procedure involved a SSMCC crosslinker molecule between the surface and the attached biomolecules. This adds both complexity to the assay and distance between the molecule and the transducer. The latter could negatively influence electronic read-out, since distance is inversely correlated with detection sensitivity. This procedure is outlined in Figure 11B. Gu and coworkers functionalised p-type diamond with a poly-aniline (PANI)/poly-acrylic acid (PAA) composite polymer films using CV. The p-type diamond working electrode was treated with the aniline and PAA monomeric solution, and by potential cycling the monomers were polymerised onto the electrode. In a final step, NH_2_-modified ssDNA was covalently attached to the exposed COOH-groups of the PANI/PAA polymeric film by 1-ethyl-3-(3-dimethylaminopropyl)-carbodiimide (EDC). The selective hybridisation was verified by fluorescence and impedance spectroscopy. The linear range of target ssDNA detection was 200 to 50 nM, with a detection limit of 20 nM [32].

##### Photochemical biofunctionalisation

Undoped, H-terminated NCD surfaces were covered with trifluoroacetamide acid (TFAAD) inside a nitrogen-purged Teflon reaction chamber by Yang and coworkers. It is a 10-aminodec-1-ene molecule, protected with a trifluoroacetic acid group at one end. The other end is terminated by a C=C double bond. The chamber was sealed with a quartz window, allowing the passage of UV-light from a low-pressure mercury lamp (0.35 mW.cm^−2^ measured at the sample surface) for 12 h. This illumination process caused a covalent bond to be formed between the TFAAD and the H-terminated NCD, exposing the trifluoro-acetic acid groups at the NCD surface. X-ray Photo-electron Spectroscopy (XPS) analysis confirmed the formation of a dense TFAAD monolayer [7]. The exact mechanism of this photochemical reaction has not been completely elucidated, but Nichols and coworkers suggest two possible scenarios. Both of them involve the formation of TFAAD anions by UV-induced photo-emitted electrons from the NCD. In one scenario, these anions react directly with the H atoms on the NCD surface. In the other scenario, the anions abstract H atoms from the surface, creating surface C dangling bonds that can covalently bind with other TFAAD anions that are present [33]. After TFAAD attachment, the trifluoro-acetic acid groups were removed by immersion into a hydrochloric acid (HCl)/methanol solution, forming NH_2_-modified NCD surfaces. These were subsequently exposed to the heterobifunctional cross-linker molecule SSMCC. SH-modified ssDNA molecules could then be linked to the SSMCC in the same way as described above. Figure 11C represents the reaction steps that were employed. The functional activity of these ssDNA molecules was confirmed by the fluorescent detection of selective hybridisation with target ssDNA, and later, on p-type NCD, with impedance spectroscopy [34]. However, the use of a permanent crosslinker allows the same remarks as with electrochemical biofunctionalisation.

The above mentioned photochemical modification procedure involved crosslinker molecules between the surface and the attached biomolecules. Hence, the same remarks are valid as with electrochemical biofunctionalisation. Our group developed a prototype of a DNA-sensor based on NCD, being sufficiently flat to make polishing unnecessary and offering an enlarged surface area for biomodification due to the smaller grain structure. Moreover, the devised procedure for the covalent attachment of DNA is only a two-step photochemical method using a flexible linker and a zero-length crosslinker (Figure 11D) [30,31]. Undoped, H-terminated NCD was immersed in a fatty acid molecule, 10-unedecenoic acid (10-UDA), consisting of a reactive C=C double bond on one end, and a COOH-group on the other end. A 20 h illumination with UV-light (2.5 mW·cm^−2^) also caused a covalent bond to be formed between the fatty acid and the H-terminated NCD, yielding a COOH-modified NCD surface. NH_2_-modified DNA could then be reacted with these COOH-groups via EDC, resulting in covalently bound DNA molecules to NCD through a NH bond. The procedure proved to be easy, reproducible, and highly efficient. The presence of the fatty acid linker molecule offers mobility to the attached DNA, increasing their availability for hybridisation reactions. Moreover the EDC crosslinker did not remain present in the eventual amide bond, resulting in a smaller distance between NCD and DNA. The functional activity of this DNA was verified with PCR and gelelectrophoresis [30], and later with impedance spectroscopy [35].

## 3. Biosensor Classification

As already mentioned briefly, biosensors can be classified according to two systems. One can classify biosensors according to their biological selectivity for certain targets, inferred by their attached biological receptor molecules. The broad range of possible biological receptor molecules and the correlated biosensor types are summarised in Table 2.

On the other hand, different transducer materials allow different physical signal transduction principles. Hence, another way to catalogue biosensors is according to the mode of signal transduction. The most frequently measured parameters accompanying the biological target recognition reaction and the concomitant transduction-based biosensor terminology are reviewed in Table 3. In the following subsections, and in Table 4, an overview of DNA-based biosensors with different transduction mechanisms is given.

### 3.1. Electrochemical Transduction

An electrochemical biosensor is a biosensor based on an electrochemical transducer. They are by far the most commonly used for clinical analysis and the most frequently cited in literature. These types of measurements are performed in an electrochemical cell equipped with a maximum of three electrodes. At the working electrode the recognition reaction of the analyte takes place. It can be constructed from a variety of materials, such as Pt, Au, glassy carbon, palladium, Si, and of course diamond, because of its favourable electronic properties even surpassing those of the popular Si (see Table 1 and Section 2.2). The reference electrode, usually Ag/AgCl or a saturated calomel electrode (SCE), has a known and constant potential. A counter electrode carries current flow away from the reference electrode. Electrochemical measurements can be subdivided into several categories [1,36].

#### 3.1.1. Amperometric

In amperometry, current generated in the electrochemical cell during the redox reaction of electro-active analytes is measured while the applied potential driving the redox reaction is kept constant. Usually a three-electrode setup is used. The reference electrode monitors fluctuations in voltage and hence, current, between the working and the counter electrode. A potentiostat then adjusts the current flow between these two electrodes to keep the voltage at a constant level.

The working electrode is modified with the bioreceptor molecule of choice, such as ssDNA, enzymes or antibodies. The electrolyte contains the target to be quantified. This target can be an intrinsically electro-active species, such as complementary ssDNA in a DNA-based biosensor, it can cause the formation of electro-active species, such as an enzyme substrate in an enzymatic sensor, or it can carry an enzymatic label that forms the electro-active species. In first generation amperometric sensors, the redox reaction involves the depletion of O_2_ to form H_2_O_2_. This H_2_O_2_ is then oxidised or reduced at the working electrode. When the working electrode is set at a positive potential relative to the reference electrode, it will support an oxidation reaction and it is referred to as the anode of the electrochemical cell. When the working electrode is set at a negative potential relative to the reference electrode, it will support a reduction and it is referred to as the cathode of the electrochemical cell. The current flow in the electrochemical cell that accompanies this oxidation or reduction reaction is measured, and is directly proportional to the target concentration. In second generation sensors, soluble electron mediators participate in the redox reaction. They, instead of H_2_O_2_, will become oxidised or reduced at the working electrode, generating the current to be detected. In a third generation sensor, the redox current between the target and the working electrode is directly detected, without the need for H_2_O_2_ or redox mediators.

Cyclic Voltammetry (CV) is a popular amperometric technique used to measure current in an electrochemical cell associated with the redox reaction of a certain analyte. In contrast to the general amperometric principle explained above, the potential of the working electrode relative to the reference electrode is not fixed, but varies linearly over time. The measurement starts at a potential where no redox reaction occurs. When the potential is swept to anodic values, the electro-active species will become oxidised at a certain potential. When all of the species are oxidised and the potential is subsequently swept back to more cathodic voltages, the electro-active species will undergo a reduction at a certain voltage. The rate at which the voltages are swept is also an important parameter. CV rapidly provides considerable information on the thermodynamics of redox reactions and electron transfer processes, and offers a rapid localisation of redox potentials of the electro-active species.

Amperometric DNA-based biosensors make use of their intrinsically electro-active bases, guanine (G) and adenine (A). Probe ssDNA is attached to the working electrode surface, and hybridisation is detected by means of redox mediators. The G and/or A residues in the DNA and the redox mediators participate in a redox reaction, followed by the re-oxidation of the mediator at the anodic electrode. The current is proportional to the amount of G and/or A, being larger in dsDNA. Direct detection of the G and/or A bases without the use of a redox mediator is generally considered to be too insensitive.

Ye and coworkers constructed an electrochemical biosensor for the detection of Hepatitis B virus (HBV) DNA based on CV. They covalently immobilised ssDNA through their 3′ OH-end to the COOH-groups of thioglycolic acid SAMs on a Au working electrode. Cyclic voltammograms were obtained from −0.3 V to 0.7 V vs. SCE, in the presence of the redox mediator ferrocenium hexafluorophosphate (FcPF_6_). They reached a detection limit of 0.2 fM of PCR amplified HBV DNA. As a selectivity control, aspecific calf thymus DNA was used [37].

Although the fact that a very high sensitivity is reached with DNA molecules that come straight out of PCR, some disadvantages are associated with the choice of transducer material. Gold causes substantial background current in CV [8] and makes for a rather unstable biomolecule-gold interface [7]. This approach can also not be termed label-free because of the requirement of an electron mediator.

#### 3.1.2. Coulometric

Coulometry is an electrochemical technique related to amperometry. In this case, the amount of charge instead of the amount of current is measured between the electrodes, related to the oxidation and reduction reactions of the electro-active analytes at the working electrode.

#### 3.1.3. Potentiometric

In potentiometry, the electrical potential difference between working and reference electrode in the electrochemical cell is measured when the current in the electrochemical cell is zero. This change in potential is directly correlated with the logarithmic analyte concentration. Usually, the potential of the working electrode varies depending on the concentration of charge or ions in the electrolyte. Hence, the most common potentiometric sensor is a pH-sensor. It is an ion-sensitive electrode (ISE) that is sensitive to the hydrogen ion (H^+^) concentration in the electrolyte. However, since a pH-sensor does not make use of a biological receptor component, this type of sensor is referred to as a chemical sensor instead of a biosensor.

Wang and coworkers utilised an enzyme-amplified method for hybridisation detection. Streptavidin-modified magnetic beads were functionalised with biotinylated ssDNA. Target ssDNA related to the BRCA1 breast cancer gene were modified with multiple copies of AP through a streptavidin-biotin bond, and exposed to the probe ssDNA-modified beads for hybridisation. After hybridisation, the AP substrate, α-naphtyl phosphate, was added and metabolised to α-naphtol. Finally, the complexes were magnetically separated from the supernatant, the latter containing the enzymatic product α-naphtol. This α-naphtol-containing supernatant was transferred to a three-electrode electrochemical cell with a carbon-nanotube working electrode, a Pt wire counter electrode and a Ag/AgCl reference electrode for potentiometric measurements of α-naphtol. A dynamic range of 3.3–20 nM was determined, and a detection limit of ∼6.6 pM. As a selectivity control, random, non-complementary DNA was used [38].

Again, the good sensitivity of this potentiometric DNA-sensor is overshadowed by the requirement of a label, complicating the setup. Moreover, no attempt was made to explore the sensitivity in terms of number of detectable mismatches. In this context of breast cancer, single nucleotide polymorphism (SNP) sensitivity in the BRCA1 gene is of utmost importance. In our research, we did focus on the establishment of SNP sensitivity.

#### 3.1.4. Conductimetric

Conductimetric devices detect changes in conductivity, or the ability to carry current, of the electrolyte between working and reference electrode in an electrochemical cell. Such changes in conductivity can arise due to changes in ionic strength of the electrolyte. Thus, it offers another way of determining the analyte concentration through the generation of ions.

Park and coworkers presented an unusual setup for the conductimetric detection of DNA hybridisation. Probe ssDNA was covalently immobilised onto a silicon/silicon dioxide (Si/SiO_2_) wafer, between two Au/tin electrodes. Longer target ssDNA was added and hybridisation occurred between probe ssDNA and one half of the target sequence. Subsequently, ssDNA-modified Au nanoparticles, complementary to the other half of the target ssDNA were added. Ag deposition to these Au nanoparticles leads to a measurable conductivity increase between the two electrodes. Target ssDNA was detected in the range of 50 nM to 500 fM. As a selectivity control, three types of 1-mismatch ssDNA were used [39].

The sensitivity obtained by these authors is due to the use of two signal amplification systems, complicating their procedure as a whole, but also the implementation of the platform into a lab-on-chip.

#### 3.1.5. Impedimetric

Electrochemical Impedance Spectroscopy (EIS) is a useful tool for label-free and real-time target detection, which will decrease cost and analysis time. In contrast, the previously summarised techniques, such as amperometry, conductimetry and potentiometry, very often required label-based signal amplification and detected their selective analytes in an endpoint configuration where ‘before target addition’ was compared to ‘after target addition’.

If an alternating (AC) voltage is applied to an electronic circuit containing capacitive and inductive elements, the complex resistance to current flow is called impedance, *Z*. An AC potential is generated over a range of frequencies between the biologically modified semiconductor working electrode and a counter electrode. The impedance is subsequently measured between these two electrodes, through the electrolyte, for each frequency in the analysed frequency range.

**Figure 12.**
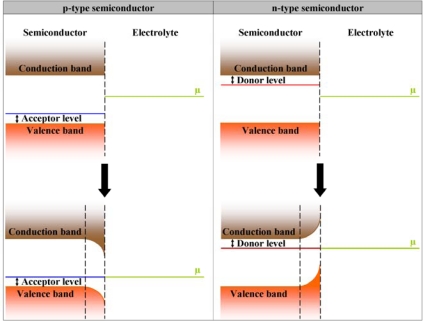
Generation of thermodynamic equilibrium in p-type (left panel) and n-type (right panel) semiconductors through downward and upward band bending, respectively.

As already mentioned in Section 2.2.1, when a semiconductor electrode is placed into contact with an electrolyte, the E_F_ of the semiconductor and the chemical potential of the electrolyte, μ, are initially not in equilibrium. Two alternative events can occur to obtain the necessary thermodynamic equilibrium, depending on the type of semiconductor. These are shown in Figure 12.

When a p-type semiconductor is placed in contact with a liquid, electrons move from the electrolyte into the semiconductor, thereby depleting the positively charged holes in the material and creating a region just below the semiconductor surface where no majority charge carriers exist. This region is called the depletion zone or the space-charge region. When no more electrons move into the semiconductor, thermodynamic equilibrium is reached between the E_F_ and μ, resulting in a downward bending of the valence and conduction bands in the p-type semiconductor.

When a n-type semiconductor is placed in contact with a liquid, electrons move from the semiconductor into the electrolyte, also decreasing the amount of majority charge carriers in the material and creating a depletion zone or space-charge region just below the semiconductor surface. At thermodynamic equilibrium, the result is an upward band bending.

Any chemical modification in the electrode-electrolyte interface, for instance the hybridisation of target ssDNA to a ssDNA-modified electrode, will alter this equilibrium, and hence the degree of band bending. In other words, the depletion zone in the semiconductor can be made wider or narrower by external events. These phenomena occurring in the semiconductor are called field-effects. A narrowing of the depletion zone corresponds to a decrease in impedance, since the obstacle for charge carriers that want to cross this space-charge region decreases. Conversely, a widening of the depletion zone corresponds to an increase in impedance [23].

When ssDNA is attached to the surface of a p-type semiconductor, their negative charges attract the holes in the semiconductor to the surface-DNA interface. The space-charge region becomes narrower, and the downward band bending becomes less steep. Additional negative charges brought about by hybridisation will increase this effect even more. The result is a decrease in impedance. When ssDNA is attached to the surface of an n-type semiconductor, their negative charges repel the electrons in the semiconductor. The space-charge region becomes wider, and the upward band bending becomes more pronounced. This effect is again amplified by hybridisation. By modelling the observed impedance effects with an electrical circuit, one can associate certain effects with changes in electrical elements, further elucidating the events at the molecular level.

**Figure 13.**
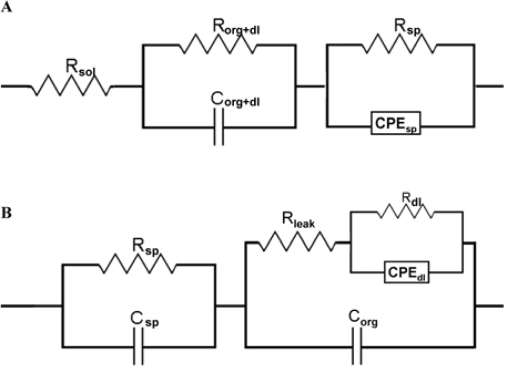
Electrical circuit models employed for the interpretation of impedance measurements. A: Model used by Yang and coworkers to interpret the effects at a DNA-modified semiconductor interface [34]. The equivalent circuit consists of a resistance R_sp_ and a constant-phase element (CPE_sp_) in parallel, representing the space-charge region in the diamond surface. The molecular layer, comprising the DNA and the linker layer, and the associated double-layer, are represented by a parallel configuration of a resistance R_org+dl_ and capacitance C_org+dl_. The resistance of the solution is represented by R_sol_. B: Model used by Cai and coworkers to interpret the effects at a DNA-modified semiconductor interface [40]. The equivalent circuit consists of a resistance R_sp_ and a capacitance C_sp_ in parallel, representing the space-charge region in the silicon surface. The capacitor C_org_ represents the molecular layer, comprising the DNA and the linker layer. A leakage resistance R_leak_, reflects the penetration of the electrolyte through the molecular layer. The electrical double-layer is represented by a CPE_dl_ and a resistance R_dl_ in parallel.

Yang and coworkers monitored selective DNA hybridisation using EIS. H-terminated NCD working electrodes of p-type nature were covalently modified with thiolated ssDNA molecules (see Section 2.3). A Pt foil and a Ag/AgCl wire were used as counter and reference electrode, respectively. They showed that measurements at open-circuit potential displayed a significant decrease in impedance at frequencies of >10^4^ Hz, even in real-time, when the NCD electrode was exposed to complementary target ssDNA, while 4-mismatch sequences were easily discriminated. By electrical circuit modelling, using the model displayed in Figure 13A, they attributed this effect to a hybridisation-induced field-effect in the NCD film [34].

Cai and coworkers performed analogous experiments on n-type Si working electrodes. They showed that hybridisation with complementary target ssDNA increased the impedance at frequencies >10^3^ Hz, illustrating nicely the opposite effects of p- and n-type electrodes. The selectivity was demonstrated with 4-mismatch sequences. By modelling with the electrical circuit shown in Figure 13B, they attributed the observed effect to a hybridisation-induced increase in the resistance of the Si film [40].

Gu and coworkers covalently immobilised NH_2_-modified ssDNA onto p-type diamond with PANI/PAA composite polymer, as mentioned in Section 2.3. A three-electrode system was used for EIS. The p-type diamond served as a working electrode, the counter electrode was a Pt wire and the reference electrode was Ag/AgCl. They observed an impedance decrease, this time in the lower frequency regions (10–100 Hz) upon complementary hybridisation, and a decrease in electron-transfer resistance to the electrode. Electric circuit modelling, using the same circuit model as Yang and coworkers, attributed this lower frequency region to reflect the polymer/molecular double-layer. They suggest that hybridisation with complementary DNA decreases the resistance and increases the capacity of the polymer, both due to an increase in ionic density at the interface. The space-charge region of the p-type diamond electrode was reflected at frequencies of ∼1,000 Hz. They found that DNA hybridisation also altered the electrical response of the electrode through a field-effect, resulting in a decreased impedance in this space-charge region. The linear range of target ssDNA detection was 50 to 200 nM, with a detection limit of 20 nM. They obtained SNP sensitivity [32].

In our group, we investigated the possibility of SNP detection on lightly p-type NCD using EIS. Probe ssDNA molecules were covalently attached to COOH-modified NCD working electrodes. The frequency-dependent change in impedance was analysed in real-time during hybridisation with complementary, 1-mismatch and non-complementary target ssDNA using the same model as Yang and coworkers SNP discrimination was possible in real-time during hybridisation in a frequency region around 1,000 Hz. Interesting was the fact that this SNP-sensitivity was also reached when analysing the denaturation reaction in real-time, in this case at the highest frequency of 1 MHz [35].

Keighley and coworkers devised an impedimetric DNA-sensor based on a Hg/Hg_2_SO_4_ reference electrode, a Pt counter electrode, and a Au working electrode. The latter was functionalised with SH-modified peptide nucleic acid (PNA) and mercaptohexanol (MCH) as a spacer molecule. Ferri/ferrocyanide was used a redox couple. Hybridisation with complementary ssDNA resulted in an increase in Z due to a higher charge-transfer resistance (R_ct_) for the redox couple as compared to non-complementary ssDNA, and a detection limit of 1 nM was reached [41].

#### 3.1.6. Field-Effect

Field-effect transistor (FET)-based biosensors offer an alternative approach for the label-free and real-time detection of changes in ionic charge concentrations in the electrolyte associated with the recognition and binding of target by electrode-immobilised biomolecules.

**Figure 14.**
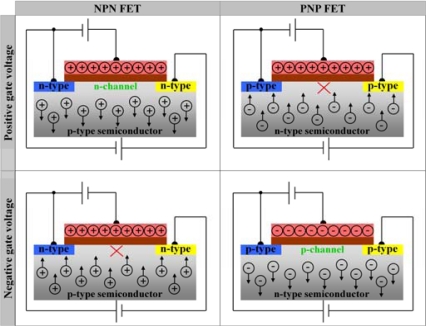
Schematic diagram of the varying source-drain current density as a function of semiconductor type and gate voltage. Top left: Applying a positive gate voltage to a n-channel FET (NPN FET) will create a conductive channel from source to drain. Bottom left: Applying a negative gate voltage to a n-channel FET (NPN FET) will block current flow between source and drain. Top right: Applying a positive gate voltage to a p-channel FET (PNP FET) will block current flow between source and drain. Bottom right: Applying a negative gate voltage to a p-channel FET (PNP FET) will create a conductive channel from source to drain.

A FET is composed of four elements: a drain, a source, a gate and a semiconducting body. Depending on the type of semiconductor used as body electrode, also two types of FET structures can exist. When the semiconducting body electrode is n-type, the source and drain electrodes are p-type (PNP or p-channel FET). In the opposite configuration, the body electrode is p-type, the source and drain electrodes are n-type (NPN or n-channel FET). The FET controls current flow from the source to drain by affecting the size and shape of the space-charge region in the semiconducting body electrode by applying a negative or positive voltage to the gate, as is shown in Figure 14.

- *Applying a negative gate voltage to an n-channel FET (NPN FET)* causes the positive charge carriers in the p-type semiconducting body electrode to become attracted to the gate electrode. This positively charged channel blocks current flow between source and drain.
- *Applying a positive gate voltage to an n-channel FET (NPN FET)* will create a conductive channel from source to drain. By attracting electrons from source and drain to the gate electrode and repelling the positive charge carriers from the p-type semiconductor body electrode further into the bulk, the resistance in the space-charge region decreases and current flow between source and drain increases.
- *Applying a negative gate voltage to a p-channel FET (PNP FET)* will create a conductive channel from source to drain. By attracting positive holes from source and drain to the gate electrode and repelling the electrons from the n-type semiconductor body electrode further into the bulk, the resistance in the space-charge region decreases and current flow between source and drain increases.
- *Applying a positive gate voltage to a p-channel FET (PNP FET)* causes the electrons in the n-type semiconducting body electrode to become attracted to the gate electrode. This negatively charged channel blocks current flow between source and drain.

From a biosensor perspective, hybridisation with target ssDNA to ssDNA immobilised onto the gate can influence the gate potential. Thus, one can measure the influence of the target recognition reaction directly on the source-drain current, or one can measure the compensatory change in gate potential necessary to keep the source-drain current at a constant level [42].

Ingebrandt and coworkers employed a FET-based technique to detect DNA-hybridisation in real-time and label-free, in a microarray setup. Si of n-type nature was used as a body electrode, and a reference electrode applied the gate voltage. The insulating gate oxide was SiO_2_. The total transistor chip consisted of 16 of these PNP FET-units. A differential approach proved the most reliable, where one of two electrically identical chips, the reference chip, is covalently modified with a non-complementary ssDNA probe, while the other chip is modified with a probe complementary to the target ssDNA. A pronounced field-effect was detected when hybridising in low-ionic strength Tris/EDTA (TE) buffer, to minimise the counter-ion screening effect that blocks the effect of charge accumulation to the electrolyte-oxide interface. Such a low-ionic buffer is however not ideal to support hybridisation reactions. Complementary DNA hybridisation will increase the amount of negative charge at the n-type semiconductor surface. This will cause a repulsion of the majority charge carriers in the n-type semiconductor, and an attraction of the holes in drain and source towards to the interface, leading to the formation of a conductive channel in the space-charge region and an increase in current flow in the p-channel. The selectivity of their FET-biosensor was, however, not high enough for SNP identification [43].

Song and coworkers devised a FET device for real-time DNA hybridisation detection based on a H-terminated diamond body electrode and a Ag/AgCl reference or gate electrode. The diamond was covalently functionalised with ssDNA molecules. Like in the work by Ingebrandt, hybridisation was most pronounced in a low-ionic buffer. The additional negative charge accompanying complementary DNA hybridisation increased the hole density in the surface accumulation layer of H-terminated diamond (see Section 2.2.1). This caused an increase in current flow through this p-type surface channel. They did succeed in SNP distinction from complementary target ssDNA at a concentration of 100 pM. However, their sensitivity decreased when monitoring hybridisation in real-time, because of the higher ionic strength of the buffer needed [44].

In summary, a FET-based detection technique is suitable for DNA hybridisation detection, but, just as with FET-based immunosensors, suffers from a rather high susceptibility to counter-ion screening effects as compared to impedimetric sensors. The solution, using hybridisation buffers of lower ionic strength, is sub-optimal for hybridisation promotion, often leading to decreased sensitivity.

### 3.2. Optical Transduction

Optical sensors make use of optical principles for the transduction of biological reactions into a readable output signal. The biological event can be detected by a change in observable optical properties, such as absorption, fluorescence, chemiluminescence or refractive index. Roughly, optical biosensors can be classified as indirect optical biosensors, requiring labels that are responsible for the optical effect, and direct optical biosensors, requiring no label and exploiting the interference of the biological interaction with light [1,36].

#### 3.2.1. Indirect

The major advantage of indirect over direct sensing is the improvement of the sensitivity of the assay, because of the presence of the label. However, this same label also leads to increased complexity and cost of the assay.

In DNA-based biosensors, the target ssDNA is often fluorescently labelled. After the hybridisation reaction to the immobilised probe ssDNA, the presence, absence or degree of fluorescence emission upon illumination of the sample with laser light of a wavelength as close as possible to the absorption maximum of the label, indicates the selectivity of the hybridisation.

Since 1995, this principle has been put into use, miniaturised and expanded to the modification of surfaces such as glass with 1,000–10,000 ssDNA probes per cm^2^ [45]. This came to be known as the DNA microarray. Probe DNA can be immobilised through simple adsorption via spotting, or through on-chip synthesis via photolithography. The latter approach has been patented by Affymetrix. Based on the type of nucleic acids that are attached to the microarray, one can distinguish between oligonucleotide microarrays and complementary DNA (cDNA) microarrays.

In oligonucleotide microarrays, short genomic ssDNA fragments are spotted on the microarray, and all of the sequences on the array can cover an entire genome. Oligonucleotide microarrays are therefore mainly used for extensive genetic profiling and mutational analysis. The arrays are hybridised with fluorescently labelled genomic DNA and evaluated with a fluorescence microscope equipped with a CCD camera. These microarrays yield absolute values of the presence or absence of each particular gene sequence on the array and therefore, the comparison of two conditions, such as a healthy control and a cancerous patient, requires the use of two separate microarrays for the parallel genotyping or mutation analysis of multiple genes.

**Figure 15.**
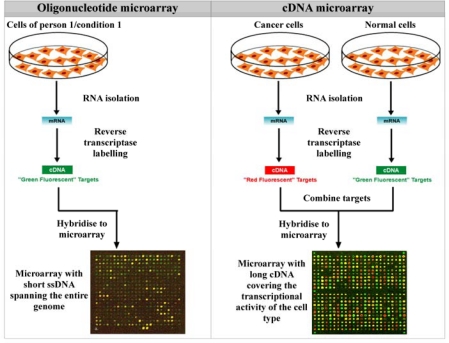
Schematic diagram of two types of microarrays. Left panel: An oligonucleotide microarray, modified with short oligonucleotide sequences covering the entire genome, gives information about the presence and absence of certain gene fragments. Each microarray is hybridised with target material of one condition. Right panel: A cDNA microarray, modified with longer cDNA sequences covering the expressional activity of a cell type, are used for gene expression analysis. Each microarray is hybridised with target material of two conditions.

In cDNA microarrays, entire cDNA molecules are spotted on the microarray, and all of the sequences can cover the expressional activity of a certain cell or tissue type. cDNA microarrays are therefore mainly used for gene expression analysis. To compare two conditions, such as the gene expression level in a certain tissue of a healthy control and gene expression level in a certain tissue of a cancerous patient, mRNA is isolated from the tissue of the healthy control and from the tissue of the cancerous patient. Both mRNA sources are labelled with a different fluorescent dye, and the array is hybridised with a mixture of both mRNA sources. The fluorescence is again evaluated with a fluorescence microscope. These microarrays compare the expression level of the two conditions for each particular gene on the array. Hence, one microarray can be used for the expression analysis of two conditions. A schematic representation of an oligonucleotide microarray and a cDNA microarray is shown in Figure 15.

Wu and coworkers developed an oligonucleotide microarray to distinguish between the Cyanine (Cy) 5-labelled pathogenic O157:H7 and Cy3-labelled non-pathogenic K12 *E. Coli* strains [46].

Schwonbeck and coworkers devised an alternative, reverse, method for oligonucleotide microarray construction, eliminating the disadvantage of a serial approach, i.e. the need for one chip per experimental condition or patient. They immobilised single-stranded and biotinylated PCR products of a well-known polymorphism of the gene for cytosolic sulfotransferase (SULT1A1*2) from 24 homozygotic individuals onto an avidin-coated microscope cover slip. Subsequently, they hybridised the array with fluorescently labelled target ssDNA, complementary to the wild-type. The final step after hybridisation involved a dissociation of the fluorescent strand from the immobilised strand. The mismatched hybrids showed a higher dissociation rate than the complementary duplexes. They even succeeded in the discrimination of heterozygotes [47].

Despite the enormous advances made in microarray technology and their widespread use in research and diagnostics because of its high-throughput construction, several disadvantages need to be mentioned. The requirement of target-labelling and the associated need for very expensive complex optics for detection make microarrays an extremely costly investment. The fact that the substrate material, usually Si, yields a rather unstable bond with biomolecules and hence allows only disposable applications only exacerbates this fact [7,8]. Also, microarrays employ an endpoint manner of detection. This implies one hybridisation condition for all of the probes attached to the surface. However, many factors, such as the type of mismatch (involving pyrimidines or pyridines), influences the hybridisation efficiency. This sort of information could be lost after a significant hybridisation time, and incorrect conclusions will be drawn. Finally, many internal controls need to be included in the microarray design for statistical analysis, leading to increased complexity and a decrease in sample throughput.

All of these problems can be circumvented using an impedimetric sensor platform. No label and no expensive machinery is needed, which will have a significant impact on cost. Moreover, since the biomolecule-diamond interface is extremely stable, repeated use is possible, also positively influencing budget. Importantly, impedimetric DNA-based biosensors allows real-time detection of hybridisation and denaturation, specifically focusing on the kinetic behaviour of different DNA molecules which is not possible with an endpoint detection. The advantage of high-throughput with microarrays is also relatively easily attainable with our setup in terms of array-functionalisation.

#### 3.2.2. Direct

In direct optical transduction, no label is required. The biological reaction itself interferes with the optical properties of the system in one way or another, which becomes the subject of detection.

Since its development in the 1980s as a method for interrogation of thin films and biological and chemical interactions, Surface Plasmon Resonance (SPR) has become a powerful technique to measure biomolecular interactions in real-time in a label-free environment. As already mentioned briefly in Section 2.2.2, when monochromatic polarised light hits an interface of two transparent media, such as a glass prism and a buffer solution, from the side of the media with the highest refractive index (glass prism), the light is partly reflected and partly refracted towards the plane of the interface. Above a certain incidence angle, all of the light is reflected and none of it is refracted. This phenomenon is called Total Internal Reflection (TIR).

In SPR, the glass prism is coated with a Au film. In conducting metals, such as Au, the free conduction electrons form periodic oscillations, called plasma waves. Like every periodic electromagnetic wave, this can also be described in a particle fashion. Like photons and phonons are the particle names for light and sound waves, respectively, a plasmon is the particle name for the plasma wave. Surface plasmons are those plasmons that are confined to the surface of the metal. They occur at the interface of the Au surface and the buffer. These plasmons create an electric field that extends about 100 nm both into the buffer solution and into the Au film and glass prism. This electrical field is called an evanescent wave, because it decays exponentially with distance. When the incident light beam has the correct incidence angle within TIR, surface plasmon resonance occurs. At this so-called ‘resonance angle’, θ, the photons in the light beam have a momentum (vector with magnitude and direction) equal to the momentum of the surface plasmons, and the photons are converted into plasmons. In other words, optical energy is coupled into the Au surface. As a result, reflection is decreased at this resonance angle.

Any change in the composition of the material at the interface between the Au and the buffer will alter the momentum of the surface plasmons, and their associated evanescent wave. As a consequence, SPR no longer occurs at the previous incidence angle, and a SPR shift takes place. The shift in the resonance angle is directly proportional to the change in mass at the Au surface, for instance during DNA hybridisation [48,49]. A well known commercial system employing SPR is the BIAcore™.

Wang and coworkers investigated the selectivity of covalently immobilised thiolated ssDNA probes onto the Au films of two commercially available SPR-based biosensors: SPREETA™ and BIAcore™. For the BIAcore™ system, complementary target ssDNA was efficiently detected in real-time in a range between 0 and 500 nM, with a linear range up to 25 nM. The limit of detection was determined to be 2.5 nM. With the SPREETA™ system, complementary target ssDNA was efficiently detected in real-time in a range between 0 and 1,000 nM, with a limit of detection of 10 nM. However, the effect of non-complementary DNA was not studied [50].

Although SPR is another method for real-time and label-free detection of molecular interaction, the restraint for Au substrates could collide with the requirement of interface stability. Moreover, implementation of SPR sensing into a point-of-care application will be unlikely in the short term.

### 3.3. Piezo-Electric Transduction

Since the antigen-recognition by immobilised antibodies, and the DNA hybridisation to immobilised ssDNA molecules increases the mass of the attached complex, mass-sensitive transducers were developed for biosensing. Although SPR also exploits the change in mass during a biological recognition event, that detection principle relies on an optical technique.

In 1917, it was shown that quartz crystals could be used as transducers of ultrasound, sounds with a higher frequency than the upper limit of human hearing. Since 1934, AT-cut quartz crystals have been used in the construction of Quartz Crystal Microbalances (QCM). Such an AT-cut quartz plate is sandwiched between two Au electrodes. Because of this mechanical compression, or stress, the quartz obtains an electrical charge due to the piezo-electric effect. When the electrodes are connected to an oscillator and an AC voltage is applied, the quartz crystal starts to oscillate and resonates at a frequency proportional to the piezo-electric thickness. It was observed that the resonant frequency of the quartz decreases upon addition of mass. In 1959, Sauerbrey demonstrated that this frequency shift is proportional to the added mass, and formulated this relationship in the Sauerbrey equation, given in (1) [51]:

(1) $$\Delta F=-\frac{{2F}_{0}^{2}\Delta m}{A\sqrt{\mu_{q}\;\rho_{q}}}$$

In this equation, Δ*m* is the added mass, *F*_0_ is the resonant frequency of the crystal, *A* is the active area of the crystal between the electrodes, *ρ_q_* is the density of quartz, and *μ_q_* is the shear modulus of quartz. Frequency measurements are easily made with high precision, making it an ideal tool to measure small masses, such as DNA hybridisation in real-time and in a label-free fashion. But, the prequisite of a quartz, being Si, could compromise interface stability. Also, its temperature sensitivity and difficult miniaturisation limit its widespread use.

Feng and coworkers immobilised thiolated ssDNA molecules onto Au-coated 9 MHz AT-cut quartz crystals in a mixed self-assemby process together with the thiolated spacer molecule, mercaptopropionic acid. Hybridisation comprised two steps. First, target ssDNA was hybridised, with one half fully complementary to the immobilised probe ssDNA and the other half fully complementary or displaying one mismatch to a biotinylated detection probe. Secondly, the biotinylated detection probe was hybridised to the remaining single-stranded part of the target DNA. When the target ssDNA does not perfectly match the detection probe, the nick between the detection probe and the immobilised probe will not be sealed by the high-fidelity *E. Coli* ligase.

In case of a perfectly matched complex, this ligase will form a proper duplex. Subsequent denaturation by heating resulted in a biotinylated, immobilised ssDNA probe in the complementary condition. When HRP conjugated to streptavidin was added, the streptavidin bound to the biotin label. HRP oxidised 3,3-diaminobenzidine (DAB) to an insoluble product in the presence of H_2_O_2_. This product precipitated onto the QCM and caused a mass amplification. However, in the case of one mismatch, the target ssDNA and the biotinylated detection probe both detached from the immobilised ssDNA probe during denaturation. The downstream amplification reaction hence did not take place. This approach proved applicable for quantification of the target ssDNA. The frequency response was linear in the range between 0.7–100 nM, with a detection limit of 100 pM [52]. Although sensitive, this method is rather complex and not real-time nor label-free.

## 4. Summary and Conclusions

In conclusion, let it be clear that biosensor research is a vast topic. The options for transduction principles and construction concepts are widespread, and depend largely on the type of target recognition and research questions involved. However, since a significant application area for biosensors is clinical diagnostics, this poses certain desires and constraints concerning construction and detection principles. The devices need to be fast, relatively cheap, portable, sensitive, specific and stable to allow a point-of-care application and repeated use. Many biosensor concepts described in literature do not comply with one or more of these features. Optical techniques require expensive machinery and operate in an endpoint setting. This increases size, cost and analysis time, restricting their use in bedside diagnostics. QCM is a very sensitive mass-detection technique that can be used to detect DNA. However, despite the fact that the technique can operate in real-time, which decreases analysis time greatly, it is quite sensitive to temperature fluctuations and poses limitations for miniaturisation and arraying. Electrical transduction however, such as impedance spectroscopy, is sensitive, it can detect DNA in real-time, and it does not require expensive equipment. Often, semiconducting electrode materials such as Si are used, allowing the devices to be miniaturised. But the interface between Si and biomolecules suffers greatly from hydrolysis in electrolytes, leading to signal drift and instability, limiting them to a disposable use. Diamond can replace Si in an otherwise very promising detection technique. Its wide electrochemical window makes it an ideal electrode, doping gives it semiconducting properties, its inertness makes it resistant to the most harsh environments, and the attachment of biomolecules through C-C-bonds creates a stable bio-interface. Because of these reasons, a lot of research effort is dedicated to the exploration of diamond as a substrate material in impedimetric DNA-sensors. However, the reproducibility of synthetic diamond growth is still an important issue to keep in mind.

It is clear that the groundwork has been laid for obtaining reliable and sensitive sensing platforms for DNA. However, many demands will need to be met and many factors taken into consideration in order for them to be applicable in routine clinical diagnostics. First of all, a prerequisite for all types of sensors for use in routine diagnostics and research is their compatibility with ‘real-life’ samples. DNA-sensors will need to retain their sensitivity and specificity when challenged with patient PCR material. Secondly, they will also need to be miniaturised in order to be portable and easy to handle, but also to decrease analysis time down to the minute-scale. Only with extensive interdisciplinary expertise, these new inroads towards biosensor development will allow the establishment of new and exciting biosensing platforms.

## Figures and Tables

**Table 1.** Overview comparing the physical properties of three semiconductors: diamond, Si, and Ge.

**Property**	**Diamond**	**Silicon**	**Germanium**
Thermal expansion (×10^−6^.K^−1^)	1.1	2.6	5.57
Band gap (eV)	5.47	1.12	0.66
Carrier mobility (cm^2^.V^−1^.s^−1^)			
→ electron	2,200	1,500	3,900
→ hole	1,600	475	1,900
Breakdown voltage (× 10^5^.V.cm^−1^)	100	3	1
Dielectric constant	5.5	11.9	16.2
Resistivity (Ω.cm)	10^13^	10^3^	46–60
Thermal conductivity (W.cm^−1^.K^−1^)	9–23	1.68	0.599
Refractive index	2.42	3.5	4
Hardness (kg.mm^−2^)	8,000	1,150	780

**Table 2.** Biosensor classification based on the type of biological receptor molecule.

**Biomolecule**	**Type biosensor**
Enzyme	Catalytic or enzyme biosensor
Affinity-complex forming biomolecules (membrane receptor, aptamer, protein, antibody, …)	Affinity-based biosensor
→ antibody	→ immunosensor
→ DNA	→ DNA-based biosensor
Cell	Whole-cell biosensor

**Table 3.** Biosensor classification based on the transduction principle.

**Measured parameter**	**Type biosensor**
Electrochemistry	Electrochemical biosensor
→ current	→ amperometric biosensor
→ charge	→ coulometric biosensor
→ voltage	→ potentiometric biosensor
→ conductivity	→ conductometric biosensor
→ impedance	→ impedimetric biosensor
→ field-effect	→ field-effect transistor-based biosensor
Optics	Optical biosensors
→ absorbtion	→ colorimetric biosensor
→ chemiluminescence	→ chemiluminescent biosensor
→ fluorescence (FRET*, reporter genes)	→ fluorescent biosensor (cell-, array-based)
→ refractive index	→ Surface Plasmon Resonance biosensor
Mass	Piezo-electric biosensor

**Table 4.** Overview of biosensors, classified according to the transduction principle and biological receptor molecule, including key measurement parameters.

**Bioreceptor**	**Transduction**	**Substrate**	**Target**	**SNP detection?**	**Limit of Detection**	**Ref.**
	***Electrochemical***					
HBV ssDNA	amperometric (CV)	Au	HBV dsDNA amplicons	No	2 fM	[37]
Biotinylated BRCA 1 ssDNA	potentiometric	magnetic beads	AP-BRCA 1 ssDNA	No	6.6 pM	[38]
SH-ssDNA	conductimetric	Si/SiO_2_	Au-ssDNA	No	50 nM	[39]
SH-ssDNA	impedimetric	p-type NCD	ssDNA	No	Undetermined (5 μM used)	[7]
SH-ssDNA	impedimetric	n-type Si	ssDNA	No	Undetermined (3 μM used)	[40]
NH_2_-ssDNA	impedimetric	p-type PCD	ssDNA	Yes	20 nM	[32]
NH_2_-ssDNA	impedimetric	p-type NCD	ssDNA	Yes	Undetermined (4 μM used)	[35]
SH-ssPNA	impedimetric	Au	ssDNA + ferri/ferrocyanide	No	1 nM	[41]
NH_2_-ssDNA	field-effect	n-type Si	ssDNA	No	Undetermined (3 μM used)	[43]
NH_2_-ssDNA		p-type PCD	ssDNA	Yes	100 pM	[44]
	***Optical***					
*E. Coli* ssDNA	fluorescent	oligonucleotide array	- O157:H7 Cy5- ssDNA - K12 Cy 3- ssDNA	No	Undetermined (2–3 μg used)	[46]
SULT1A1*2 ssDNA	fluorescent	oligonucleotide array	SULT1A1*2 ssDNA	No	Undetermined (500 nM used)	[47]
SH-ssDNA	SPR	Au	ssDNA	No	- BIAcore™: 2.5 nM - SPREETA: 10 nM	[50]
	***Piezo-electric***					
SH-ssDNA	QCM	Au coated quartz	ssDNA + detection probe	Yes	100 pM	[52]

## References

[1] Gorton L.O. (2005). Comprehensive Analytical Chemistry.

[2] Häussling L., Michel B., Ringsdorf R., Rohrer H. (1991). Direct observation of streptavidin specifically adsorbed on biotin-functionalized self-assembled monolayers with the scanning tunneling microscope. Angew. Chem. Int. Ed.

[3] Rasmussen S.R., Larsen M.R., Rasmussen S.E. (1991). Covalent immobilization of DNA onto polystyrene microwells: The molecules are only bound at the 5′ end. Anal. Biochem.

[4] Hashimoto K., Ito K., Ishimori Y. (1994). Sequence-specific gene detection with a gold electrode modified with DNA probes and an electrochemically active dye. Anal. Chem.

[5] Strother T., Cai W., Zhao X.S., Hamers R.J., Smith L.M. (2000). Synthesis and characterization of DNA modified silicon (111) surfaces. J. Am. Chem. Soc.

[6] Kremsky J.N., Wooters J.L., Dougherty J.P., Meyers R.E., Collins M., Brown E.L. (1987). Immobilization of DNA via oligonucleotides containing an aldehyde or carboxylic acid group at the 5′ terminus. Nucleic Acids Res.

[7] Yang W., Auciello O., Butler J.E., Cai W., Carlisle J.A., Gerbi J.E., Gruen D.M., Knickerbocker T., Lasseter T.L., Russell J.N., Smith L.M., Hamers R.J. (2002). DNA-modified nanocrystalline diamond thin-films as stable, biologically active substrates. Nat. Mater.

[8] Nebel C.E., Shin D., Rezek B., Tokuda N., Uetsuka H., Watanabe H. (2007). Diamond and biology. J. R. Soc. Interface.

[9] Davidson J.L. (1994). Synthetic Diamond: Emerging CVD Science and Technology.

[10] Wolfe C.M., Holonyak N., Stillman G.E. (2007). Physical properties of semiconductors.

[11] Millan K.M., Spurmanis A.J., Mikkelsen S.R. (1992). Covalent immobilization of DNA into glassy-carbon electrodes. Electroanalysis.

[12] Pierson H.O. (1993). Handbook of Carbon, Graphite, Diamond and Fullerenes.

[13] Zaitsev A.M. (2001). Optical Properties of Diamond: A Data Handbook.

[14] Wilks J., Wilks E. (1991). Properties and Applications of Diamond.

[15] Bundy F.B., Hall H.T., Strong H.M., Wentorf R.H. (1955). Man made diamonds. Nature.

[16] Lee S.T., Lin Z., Jiang X. (1999). CVD diamond films: nucleation and growth. Mater. Sci. Eng.

[17] May P.W. (2000). Diamond thin films: a 21st-century material. Phil. Trans. R. Soc. Lond. A.

[18] Liu H., Dandy D.S. (1993). Diamond Chemical Vapour Deposition: Nucleation and Early Growth Stages.

[19] Williams O.A., Nesládek M. (2006). Growth and properties of nanocrystalline diamond films. Phys. Stat. Sol.

[20] Granger M.C., Xu J., Strojek J.W., Swain G.M. (1999). Polycrystalline diamond electrodes: basic properties and applications as amperometric detectors in flow injection analysis and liquid chromatography. Anal. Chim. Acta.

[21] Maier F., Riedel M., Mantel B., Ristein J., Ley L. (2000). Origin of surface conductivity in diamond. Phys. Rev. Lett.

[22] Nebel C.E., Rezek B., Shin D., Watanabe H. (2006). Surface electronic properties of H-terminated diamond in contact with adsorbates and electrolytes. Phys. Stat. Sol.

[23] Chakrapani V., Angus J.C., Anderson A.B., Wolter S.D., Stoner B.R., Sumanasekera G.U. (2007). Charge transfer equilibria between diamond and an aqueous oxygen electrochemical redox couple. Science.

[24] Koizumi S., Kamo M., Sato Y., Ozaki H., Inuzuka T. (1997). Growth and characterization of phosphorous doped (111) homoepitaxial diamond thin films. Appl. Phys. Lett.

[25] Davis R.F. (1992). Diamond Films and Coatings: Development, Properties and Applications.

[26] Mumm D.R., Faber K.T., Drory M.D., Gardinier C.F. (1993). High-temperature hardness of chemically vapor-deposited diamond. J. Am. Ceram. Soc.

[27] Takahashi K., Tanga M., Takai O., Okamura H. (2000). DNA bonding to diamond. Bio. Ind.

[28] Ushizawa K., Sato Y., Mitsumori T., Machinami T., Ueda T., Ando T. (2002). Covalent immobilization of DNA on diamond and its verification by diffuse reflectance infrared spectroscopy. Chem. Phys. Lett.

[29] Wang J., Firestone M.A., Auciello O., Carlisle J.A. (2004). Functionalization of ultrananocrystalline diamond films by electrochemical reduction of aryldiazonium salts. Langmuir.

[30] Christiaens P., Vermeeren V., Wenmackers S., Daenen M., Haenen K., Nesladek M., vandeVen M., Ameloot M., Michiels L., Wagner P. (2006). EDC-mediated DNA attachment to nanocrystalline CVD diamond films. Biosens. Bioelectron.

[31] Vermeeren V., Wenmackers S., Daenen M., Haenen K., Williams O.A., Ameloot M., Vande V.M., Wagner P., Michiels L. (2008). Topographical and functional characterization of the ssDNA probe layer generated through EDC-mediated covalent attachment to nanocrystalline diamond using fluorescence microscopy. Langmuir.

[32] Gu H., Su X., Loh K.P. (2005). Electrochemical impedance sensing of DNA hybridization on conducting polymer film-modified diamond. J. Phys. Chem. B.

[33] Nichols B.M., Butler J.E., Russell J.N., Hamers R.J. (2005). Photochemical functionalization of hydrogen-terminated diamond surfaces: a structural and mechanistic study. J. Phys. Chem. B.

[34] Yang W., Butler J.E., Russell J.N., Hamers R.J. (2004). Interfacial electrical properties of DNA-modified diamond thin films: intrinsic response and hybridization-induced field effects. Langmuir.

[35] Vermeeren V., Bijnens N., Wenmackers S., Daenen M., Haenen K., Williams O.A., Ameloot M., van de Ven M., Wagner P., Michiels L. (2007). Towards a real-time, label-free, diamond-based DNA sensor. Langmuir.

[36] D'Orazio P. (2003). Biosensors in clinical chemistry. Clin. Chim. Acta.

[37] Ye Y.K., Zhao J.H., Yan F., Zhu Y.L., Ju H.X. (2003). Electrochemical behavior and detection of hepatitis B virus DNA PCR production at gold electrode. Biosens. Bioelectron.

[38] Wang J., Kawde A.N., Jan M.R. (2004). Carbon-nanotube-modified electrodes for amplified enzyme-based electrical detection of DNA hybridization. Biosens. Bioelectron.

[39] Park S.J., Taton T.A., Mirkin C.A. (2002). Array-based electrical detection of DNA with nanoparticle probes. Science.

[40] Cai W., Peck J.R., van der Weide D.W., Hamers R.J. (2004). Direct electrical detection of hybridization at DNA-modified silicon surfaces. Biosens. Bioelectron.

[41] Keighley S.D., Estrela P., Li P., Migliorato P. (2008). Optimization of label-free DNA detection with electrochemical impedance spectroscopy using PNA probes. Biosens. Bioelectron.

[42] Young H.D., Freedman R.A. (2000). University Physics.

[43] Ingebrandt S., Offenhäusser A. (2006). Label-free detection of DNA using field-effect transistors. Phys. Stat. Sol.

[44] Song K.S., Zhang G.J., Nakamura Y., Furukawa K., Hiraki T., Yang J.H., Funatsu T., Ohdomari I., Kawarada H. (2006). Label-free DNA sensors using ultrasensitive diamond field-effect transistors in solution. Phys. Rev. E. Stat. Nonlin. Soft. Matter Phys.

[45] Schena M., Shalon D., Davis R.W., Brown P.O. (1995). Quantitative monitoring of gene expression patterns with a complementary DNA microarray. Science.

[46] Wu C.F., Valdes J.J., Bentley W.E., Sekowski J.W. (2003). DNA microarray for discrimination between pathogenic 0157:H7 EDL933 and non-pathogenic *Escherichia Coli* strains. Biosens. Bioelectron.

[47] Schwonbeck S., Krause-Griep A., Gajovic-Eichelmann N., Ehrentreich-Forster E., Meinl W., Glatt H., Bier F.F. (2004). Cohort analysis of a single nucleotide polymorphism on DNA chips. Biosens. Bioelectron.

[48] Bernhard B., Lengeler B. (1978). Electronic Structure of Noble Metals and Polariton-Mediated Light Scattering.

[49] Flanagan M.T., Pantell R.H. (1984). Surface plasmon resonance and immunosensors. Electron. Lett.

[50] Wang R., Tombelli S., Minunni M., Spiriti M.M., Mascini M. (2004). Immobilisation of DNA probes for the development of SPR-based sensing. Biosens. Bioelectron.

[51] Sauerbrey G. (1959). Verwendung von schwingquarzen zur wägung dünner schichten und zur mikrowägung. Z. Phys.

[52] Feng K., Li J., Jiang J.H., Shen G.L., Yu R.Q. (2007). QCM detection of DNA targets with single-base mutation based on DNA ligase reaction and biocatalyzed deposition amplification. Biosens. Bioelectron.

